# Antihypertensive Effects of Aqueous Extract of *Ricinodendron heudelotii* (Baill.) Pierre (Euphorbiaceae) in Wistar Rat

**DOI:** 10.1155/2022/3305733

**Published:** 2022-11-01

**Authors:** Jacquy Joyce Wanche Kojom, Calvin Zangueu Bogning, Edwige Laure Nguemfo, Christelle Stéphanie Sonfack, Edwige Laure Lappa, Gisèle Etamé Loé, Eulogio José Llorent-Martínez, Alain Bertrand Dongmo

**Affiliations:** ^1^Department of Animal Biology and Physiology, Faculty of Sciences, University of Douala, P.O. Box 24157, Doual, Cameroon; ^2^Department of Biological Sciences, Faculty of Medicine and Pharmaceutical Science, University of Douala, P.O. Box 24157, Doual, Cameroon; ^3^Department of Physical and Analytical Chemistry, Faculty of Experimental Sciences, University of Jaén, Campus Las Lagunillas, E-23071 Jaén, Spain

## Abstract

*Ricinodendron heudelotii* stem bark is commonly used in Cameroonian traditional medicine to treat cardiovascular diseases such as hypertension. The present study was designed to investigate the antihypertensive and antioxidant properties of the aqueous extract of *Ricinodendron heudelotii* in salt-induced hypertensive rats. Analysis by HPLC-ESI-Q-TOF-MS was used to identify various chemical components of the extract. A total of thirty rats were used for each test. High-salt hypertension was induced in rats by oral administration of NaCl for 12 weeks. Mean blood pressure (MBP) and heart rate (HR) were monitored by noninvasive methods. Oral administration of *Ricinodendron heudelotii* significantly (*p* < 0.01) reduced the increase of mean blood pressure (23.12%, 26.14%, and 24.34%) and heart rate (31.19%, 31.09%, and 26.98%), respectively, at the doses of 40, 20, and 6 mg/kg, compared to the hypertensive group. All the doses tested significantly reduced or/and ameliorated biochemical and oxidative stress parameters. Histological analysis showed that *Ricinodendron heudelotii* restored renal disorders induced by the administration of salt. The aqueous extract of *Ricinodendron heudelotii* exerts a cardioprotective effect, and the antihypertensive activity seems associated with an improvement in antioxidant status. Overall, the results justify and support the traditional use of *Ricinodendron heudelotii.*

## 1. Introduction

Several studies acknowledged that hypertension is the most prevalent trigger for cardiovascular diseases (CVDs) with other risk factors [1]. It is responsible for around 9.4% million of deaths worldwide and is the main cause of morbidity and mortality associated with CVDs [2]. The increase in hypertension prevalence is due to many factors including diets rich in sugar, high-fat processed foods, and salt [3].

Sodium is the principal cation in extracellular fluid which is essential for the physiology of the body [4]. A high-salt diet has been reported to be implicated in kidney damage, oxidative stress, and hypertension [5].

The pathogenesis of hypertension is multifactorial and involves some enzyme systems, genetic influences, and oxidative stress [6]. Preclinical studies and clinical trials have indicated that antioxidant therapy is important for the management of hypertension, using antioxidant compounds such as polyphenols [7]. Likewise, several synthetic drugs have been developed for the treatment of hypertension and most of these drugs have established better effectiveness; however, they possess several side effects [8].

Attention has been drawn towards medicinal plants which are used traditionally as potential therapeutic agents in the prevention and management of cardiovascular diseases [9].

*Ricinodendron heudelotii* (Baill.) belongs to the Euphorbiaceae family, whose species possess an array of medicinal effects. In traditional medicine, *Ricinodendron heudelotii* is used in the treatment of anemia, stomachache, cough, fever, dysentery, malaria, infertility, and antidiuretic [10–12]. In West Cameroon, the folk medical practice considers the stem bark of *R. heudelotii* as a useful remedy against cardiovascular diseases. However, there is no information regarding the effect of *R. heudelotii* stem bark extract on the management of hypertension and oxidative stress. The present study was done to evaluate the potential benefits of orally administered *Ricinodendron heudelotii* stem bark aqueous extract on hypertension and oxidative stress in high-salt-induced hypertensive rats.

## 2. Materials and Methods

### 2.1. Drugs, Chemicals, and Reagents

All chemicals, drugs, and reagents used in this investigation were of analytical grade. Enzymatic and colorimetric reagent kits for the determination of alanine aminotransferase (ALT), aspartate aminotransferase (AST), creatinine, urea, total cholesterol, HDL cholesterol, triglyceride, and total protein were obtained from SGMItalia, Italy. Sodium chloride salt (NaCl) was purchased from Polypharma (Douala, Cameroon), and amlodipine was purchased from Sigma Chemical (Germany).

### 2.2. Collection of Plant Material, Identification, and Extraction

*Ricinodendron heudelotii* was harvested in Malantouen, West Region, Cameroon in December 2019, identified by M. Tacham, a botanist. The botanical identification was made to the National Herbarium of Cameroon in comparison with the sample 19695/SRFCam. The stem bark of *Ricinodendron heudelotii* was cut out, dried in the shade, and then crushed. One hundred grams (100 g) of powder were extracted by infusion in 1 liter of boiled distilled water for 20 minutes. After filtration through Whatman filter paper N°.3, the filtrate was evaporated at 40^o^C using an oven, yielding 1.21 g powder (w/w: 1.21%).

### 2.3. Animals

Both adult male and female Wistar rats of 10–12 weeks, weighing 150 to 200 g, were randomly selected from our local colonies raised in the animal house of the Faculty of Science, University of Douala, Cameroon. All procedures were approved by the Institutional Ethics Committee of the University of Douala (N°2080 CEI-UDo/04/2020/T) according to the guidelines established for the protection of animals used in experiments.

### 2.4. Phytochemical Analysis by HPLC-ESI-Q-TOF-MS

For the analysis of compounds, 5 mg of extract were dissolved in 1 mL MeOH, filtered through 0.45 *µ*m filters, and 10 *μ*L was injected in the HPLC system. Analyses were performed in an Agilent 1200 (Agilent Technologies, Santa Clara, CA, USA) equipped with an Agilent 6530 B quadrupole-time-of-flight mass spectrometer (Q-TOF-MS). A Luna Omega Polar column C18 of 150 × 3.0 mm and 5 *μ*m particle sizes (Phenomenex, Torrance, CA, USA) with a Polar C18 Security Guard cartridge (Phenomenex) of 4 × 3.0 mm were used. The separation was performed at ambient temperature with a gradient elution program at a flow rate of 0.4 mL·min^−1^. The mobile phases consisted of water + formic acid 0.1% v/v (eluent A) and acetonitrile (eluent B). The gradient elution was 10–25% B in 0–25 min, 25% B in 25–30 min, 25–50% B in 30–40 min, 50–100% B in 40–42 min, and 100% in 42–47 min. Then, eluent B was returned to 10% with a 7 min stabilization time. To obtain the MS and MS/MS spectra, the mass spectrometer was operated in the negative and positive ion modes using an orthogonal ESI source (Agilent Dual ESI, Santa Clara, CA, USA). The parameters for MS analysis were as follows: capillary voltage, 3500 V, nebulizer pressure of 45 psi, drying gas flow rate, 10 L/min, gas temperature, 325°C, skimmer voltage, 60 V, and fragmentor voltage, 140 V. The MS and Auto MS/MS modes were set to acquire m/*z* values ranging between 50 and 1200, at a scan rate of 2 and 3 spectra per second, respectively. Agilent MassHunter Qualitative analysis software version B.06.00 was used for postacquisition data processing.

### 2.5. Experimental Study Design and Treatment

Young Wistar rats (4 weeks old) were randomly divided into two groups. Group 1, the control group (*n* = 5), received a standard diet, whereas group 2, the hypertensive group (*n* = 45), received a high-salt diet (8% salt feed) daily for 8 weeks. After two months of salt loading, only rats (25 over 45) with blood pressure higher or equal to 140/90 mmHg were selected to continue this study.

The selected animals were divided as follows. The control group (group 1) received distilled water. Group 2 (hypertensive group) received a high-salt diet (8% salt feed). Group 3 (standard group) received a high-salt diet (8% salt feed) concomitantly with amlodipine (10 mg/kg). Groups 4, 5, and 6 were experimental groups and they received a concomitantly high-salt diet (8% salt feed) and plant extract at the dose of 40, 20, and 6 mg/kg, respectively. All groups had access to diet and water throughout the duration (4 weeks) of the experiment.

### 2.6. Hemodynamic Parameter Determination

Blood pressure and heart rate were measured twice weekly for 4 weeks in awake animals by a CODA noninvasive blood pressure system (Kent Scientific Co., USA) as described by Kojom et al. 2019 [13].

### 2.7. Hepatic, Renal Function, and Lipid Profile Assessment

At the end of treatment, animals were anesthetized under intraperitoneal injection (i. p) of diazepam (10 mg/kg), which was followed by i. p of ketamine (50 mg/kg) later. The blood samples were collected on anesthetized rats by retro-orbital puncture in dry tubes and centrifuged at 3000 rpm for 15 min to obtain serum which was stored at −20°c for biochemical analysis. Commercial diagnostics kits (SGMItalia, Italie) were used to determine AST, ALT, creatinine, urea, total cholesterol (TC), high-density lipoprotein cholesterol (HDL-C), triglycerides (TG) and total protein levels. Low-density lipoprotein cholesterol (LDL-C) was calculated according to the formula [14].

### 2.8. Determination of Oxidative Stress Parameters

After blood collection, the heart, aorta (starting cranially at the carotids and brachiocephalic arteries dissecting caudally to the iliac bifurcation), liver, and kidney were rapidly dissected out and freed of fat and connective tissue. The organs have been weighed after rinsing in normal saline, and representative fragments were homogenized in Mc Even solution for the heart and aorta or in Tris-HCl 50 mM buffer solution for the liver and kidney (20%, w/v). After centrifugation at 3000 rpm for 30 min, the supernatant was obtained. Tissue levels of reduced glutathione (GSH), superoxide dismutase activity (SOD), catalase (CAT), and malondialdehyde (MDA) were assayed using a colorimetric method as described by Ellman [15], Misra and Fridovich [16], Sinha [17], and Wilbur et al. [18], respectively. Nitrite (NO) contents of the tissue were determined by the methods described by Ikeda et al. [19].

### 2.9. Histopathological Analysis

Histopathological analysis on some kidney fragments was performed as described by Kojom et al. [20]. A representative fragment of the kidney was subsequently fixed in a 10% solution of buffered formalin (pH 7.4) and enclosed in paraffin for histopathological analysis. Five-micrometer sections were obtained and colored with hematoxylin-eosin for evaluation under an optical microscope. In addition, tissue collagen deposition was performed by applying the van Gieson staining.

### 2.10. Data Analysis

Statistical analysis was carried out using SigmaStat version 3.5. The values were expressed as mean ± standard error of the mean (SEM). The differences among treatment groups were analyzed by nonparametric one-way statistical analysis of variance and Kruskal–Wallis test for multiple comparisons. A *p* value less than 0.05 was considered statistically significant.

## 3. Results

### 3.1. Characterization of Phytochemicals by HPLC-ESI-Q-TOF-MS

To the best of the authors' knowledge, this is the first report on the phytochemical composition of *Ricinodendron heudelotii.* Eleven compounds were identified or tentatively characterized in the aqueous extract. The accurate mass data and MS/MS fragmentation patterns, as well as METLIN and bibliographic search, were used for the characterization.  Table 1 contains the compounds characterized, as well as retention times, experimental (M-H)^−^ or (M+H)^+^ molecular formula, calculated mass error (ppm), and fragment ions. The negative ion mode was used for all compounds except alkaloids, which were characterized using the positive ion mode.

Compounds 1, 2, 3, 4, 5, 7, 9, 10, and 11 were identified by comparison with METLIN data. Compound 5 exhibited the same fragmentation pattern previously reported for magnoflorine [21]. Compounds 6 and 8 presented protonated molecular ions at *m/z* 342 and base peaks at *m/z* 178, consistent with tetrahydroprotoberberine alkaloids [22]. Although a quantitation could not be performed due to the lack of analytical standards, most of the bioactivity of these extracts is probably due to the presence of alkaloids, which seemed to dominate the phytochemical profile.

### 3.2. Effect of R. heudelotii on Body Weight

As shown in Table 2, the body weight increased in all groups during the treatment. However, this increase was significantly (*p* < 0.001) low in test groups as compared to control during the treatment. Rats treated with salt only showed the lowest rise in body weight after four weeks of treatment by 26.39% (*p* < 0.001) than in the control group. Thus, the percentages of growth were 27.65, 30.98, 26.51, and 29.43%, respectively for salt + extract at the doses of 40, 20, and 6 mg/kg and salt + amlodipine.

### 3.3. Effect of *R. heudelotii* on Relative Organ Weight

Chronic administration of a salt diet provoked a significant increase in the relative weight of the liver (14.90%, *p* < 0.001), kidney (20.00%, *p* < 0.001), heart (17.95%, *p* < 0.01), and aorta (28.57%, *p* < 0.05) as compared to the control group (Table 3). Administration of aqueous extract provoked an opposite effect by reducing significantly the liver, kidney, heart, and aorta relative weight at the end of treatment. At a dose of 6 mg/kg, this reduction in the aorta reached 35.71% (*p* < 0.05) as compared to the hypertensive group. Amlodipine used as the standard drug did not induce significantly the variation of the relative organ weight as compared to the hypertensive group (Table 3).

### 3.4. Effect of *R. heudelotii* on Arterial Blood Pressure and Heart Rate

#### 3.4.1. Mean Blood Pressure (MBP)

**Figure 1:**
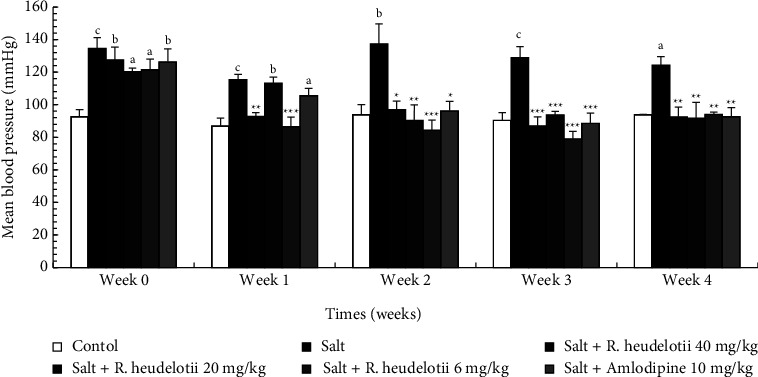
Effect of the aqueous extract of *R. heudelotii* on mean blood pressure. All values are expressed as mean ± SEM from 5 rats. ^a^*p* < 0.05, ^b^*p* < 0.01, and ^c^*p* < 0.001 compared to the control group and ^*∗*^*p* < 0.01, ^*∗∗*^*p* < 0.01, and ^*∗∗∗*^*p* < 0.001 compared to the hypertensive group. *R. heudelotii* denotes *Ricinodendron heudelotii*.

High salt intake induced a significant rise of the MBP in all groups as compared to the control before the treatment (week 0), reaching 24.49% after 4 weeks of treatment. The treatment with extract or amlodipine reduced salt-induced blood pressure from rising after four weeks of administration by 25.48% (6 mg/kg, extract) and 25.48% (amlodipine, 10 mg/kg) (Figure 1).

#### 3.4.2. Heart Rate

**Figure 2:**
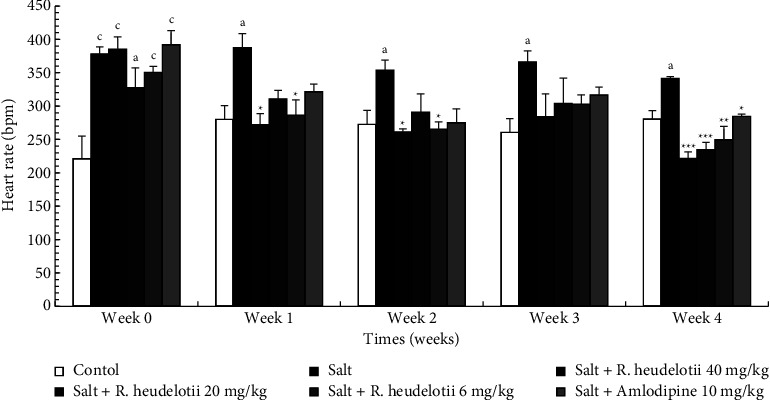
Effect of the aqueous extract of *R. heudelotii* on heart rate. All values are expressed as mean ± SEM from 5 rats. ^a^*p* < 0.05 and ^b^*p* < 0.01 compared to the control group and ^*∗*^*p* < 0.01, ^*∗∗*^*p* < 0.01, and ^*∗∗∗*^*p* < 0.001 compared to the hypertensive group. *R. heudelotii* denotes *Ricinodendron heudelotii*.

After 4 weeks of treatment, the heart rate of rats receiving salt only increased significantly by 17.77% (*p* < 0.05) as compared to the control group. Concomitant administration of salt + plant extract (40 mg/kg, 20 mg/kg, and 6 mg/kg) or amlodipine (10 mg/kg) significantly reduced this variation. At the end of treatment, the heart rate was reduced by 35.07% (*p* < 0.001), 31.15% (*p* < 0.001), 26.85% (*p* < 0.01), and 16.59% (*p* < 0.05), respectively, as compared to hypertensive group (Figure 2).

### 3.5. Effect of *R. heudelotii* Extract on the Biochemical Parameters

#### 3.5.1. Totals Protein

The total serum proteins of rats did not significantly change as compared to the control group (Table 4).

#### 3.5.2. Renal and Liver Function

High salt intake resulted in a significant increase in ALT, AST, creatinine, and urea concentrations as compared to normal rats. *R. heudelotii* (40, 20, and 6 mg/kg/day), significantly reduced the level of these parameters (Table 4).

#### 3.5.3. Lipid Profile

As shown in Table 4, total cholesterol, triglycerides, and LDL cholesterol level significantly (*p* < 0.01) increased, whereas HDL cholesterol levels significantly (*p* < 0.01) reduced in rats which received a high-salt diet compared to the control group. However, there was a significant (*p* < 0.01) drop of total cholesterol and LDL cholesterol and a significant increase in HDL cholesterol in the group treated with the extracts (20 and 6 mg/kg) when compared to the hypertensive control.

### 3.6. Effect of *R. heudelotii* Extract on Oxidative Stress Parameters

#### 3.6.1. Antioxidant Enzymes

**Figure 3:**
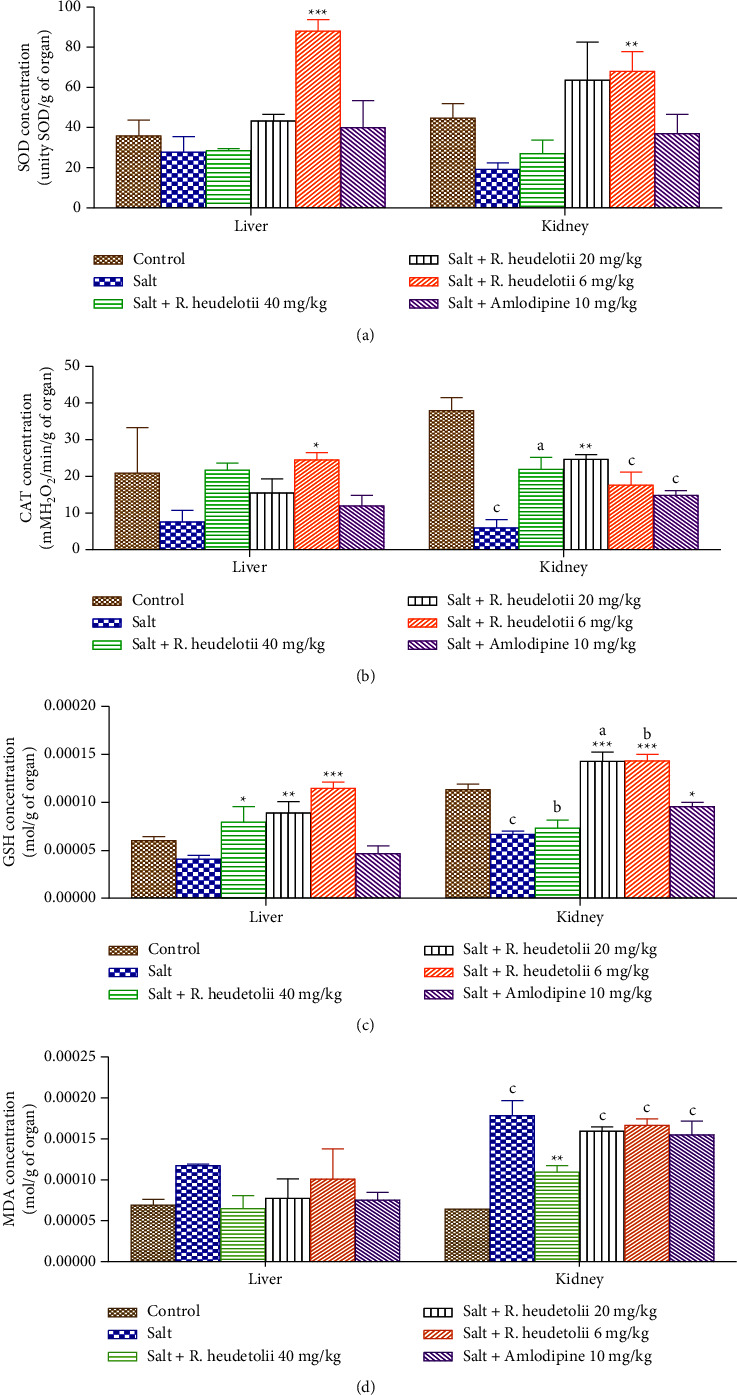
Effect of the aqueous extract of *R. heudelotii* on superoxide dismutase (a), catalase (b), glutathione reduced (c), and malondialdehyde (d) on the liver and kidney. *p* < 0.05, ^b^*p* < 0.01, and ^c^*p* < 0.001 compared to the control group and ^*∗*^*p* < 0.01, ^*∗∗*^*p* < 0.01, and ^*∗∗∗*^*p* < 0.001 compared to the hypertensive group. *R. heudelotii* denotes *Ricinodendron heudelotii*.

High salt intake by rats decreased the activity of some stress markers (SOD, CAT, and GSH) in the kidneys and liver when compared to the control group. The administration of plant extract (6 mg/kg) significantly increased the activity of SOD, CAT, and GSH in the liver and kidneys (Figures 3(a)–3(c)). Treatment of rats with amlodipine (10 mg/kg) in this assay only increased significantly (*p* < 0.05) the GSH in the kidney when compared to the hypertensive control.

#### 3.6.2. Malondialdehyde

The level of malondialdehyde (MDA) significantly (*p* < 0.01) increased in the kidneys of rats that received salt compared to the control group. Extract (40 mg/kg) inhibited the increase of MDA level in the kidney compared to control or hypertensive groups (Figure 3(d)).

#### 3.6.3. Nitrites

**Figure 4:**
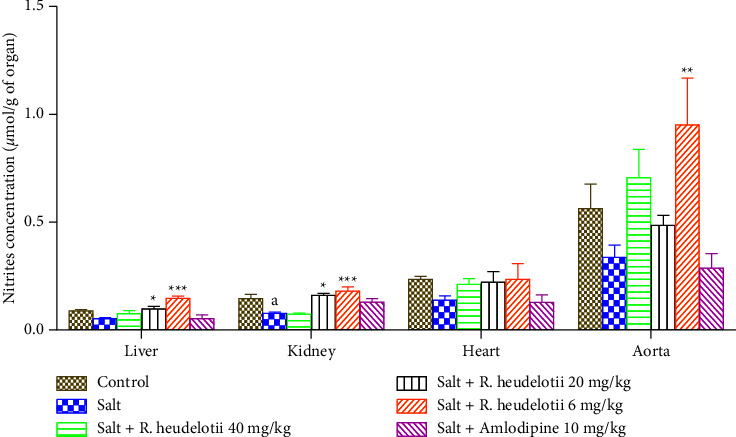
Effect of the aqueous extract of *R. heudelotii* nitrites' concentration on the liver, kidney, heart, and aorta. ^a^*p* < 0.05 compared to the control group and ^*∗*^*p* < 0.01, ^*∗∗*^*p* < 0.01, and ^*∗∗∗*^*p* < 0.001 compared to the hypertensive group. *R. heudelotii* denotes *Ricinodendron heudelotii*.

Four weeks of treatment with salt led to a significant decrease in nitrite levels in tissues as compared to the control group. The administration of extract induced a significant (*p* < 0.01) increase of nitrites' levels in the aorta (at a dose of 6 mg/kg), liver, and kidneys (at the doses of 20 and 6 mg/kg) of test groups when compared to the hypertensive group (Figure 4).

### 3.7. Histopathological examination

**Figure 5:**
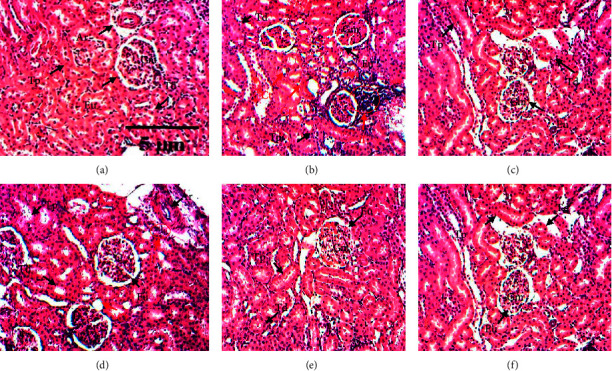
Representative photomicrographs showing the effect of the aqueous extract of *Ricinodendron heudelotii* on renal histology on salt-treated rats. Control group (a), salt group (b), salt *+ Ricinodendron heudelotii* 40 mg/kg (c), salt *+ Ricinodendron heudelotii* 20 mg/kg (d), salt *+ Ricinodendron heudelotii* 6 mg/kg (e), and salt + amlodipine 10 mg/kg (f). Ar: renal artery, Gm: glomerular, Tp: proximal tubule, Td: distal tubule, Eu: urinary space, Ct: tubular clearance, and Il: leucocytic infiltration. Sections were stained with H&E and observed at 400×.

Histopathological results of a kidney (control) showed normal and intact glomeruli, Bowman's capsule, and urinary space (Figure 5(a)). In contrast, tubular clearance and leucocytic infiltration were observed on a section of the kidney obtained from the rat treated with salt only (Figure 5(b)). The administration of the different doses of extract plant or amlodipine in animals, reduced tubular light and leucocytic infiltration compared to the hypertensive group. The administration of the doses of 40 mg/kg (Figure 5(c)) and 6 mg/kg (Figure 5(e)) significantly improved the associated lesion observed in the hypertensive group. Moreover, tubular clearance and leucocytic infiltration were absent, while urinary space was normal and renal architecture was close to the control group.

After van Gieson staining (result not presented in this study), an increase in collagen deposition in the tubular interstitial area was also shown in the hypertensive group. However, the administration of *R. heudelotii* extract showed a dose-dependent decrease in collagen deposition as compared to the hypertensive group.

## 4. Discussion

In this study, the beneficial impact of the aqueous extract of *R. heudelotii* extract on arterial blood pressure and on some biochemical and oxidative stress parameters in Wistar rats with salt-induced hypertension is shown.

The oral administration of *R. heudelotii* or amlodipine for 4 weeks significantly reduced the rise in blood pressure in salt hypertensive rats. It has been reported that excess sodium intake provoked an increase in blood pressure in rat models probably by sympathetic overactivity, through other mechanisms that affect vascular reactivity and renal function [23–25]. These results suggest that the aqueous extract of *R. heudelotii* could interfere with the mechanisms of action that contribute to increased blood pressure in a high-salt diet. Thus, the blood pressure-lowering effect of *R. heudelotii* may be due at least to the presence in the plant extract of magnoflorine and tetrahydropalmatine (THP) whose hypotensive and antihypertensive activities have been demonstrated [26, 27]. The magnoflorine may act through the muscarinic and serotonergic systems [27], while THP induces its effects through the 5-HT2 and/or D2-receptor antagonism in the hypothalamus and bradycardia in rats [26]. The presence of these two active principles in the plant extract indicates that it would act at least similarly. Moreover, the reduction of the heart rate compared to the hypertensive group observed at the end of the treatment may be due to the bradycardia effect of THP. Administration of extract protected the treated animals against the salt loading-induced increase in heart weight. Therefore, regression of heart hypertrophy could be a primary goal in antihypertensive treatment [28]. This may be one of the bases of its antihypertensive action and is an indication of the cardioprotective potential of the extract. Previous studies indicated that high-salt-induced ventricular hypertrophy in the hypertensive rat through various mechanisms including renin-angiotensin system probably by increased renin and aldosterone production [29]. Administration of extract protected the treated animals against the salt loading-induced increase in heart weight, a consequence of the antihypertensive effect of the plant, also indicating its cardioprotective properties. The citric acid present in this plant extract would strengthen this activity since it has been shown that certain organic acids have antioxidant properties and protective effects on heart disease by direct cardiomyocyte protective effects [30].

It is well known that low salt intake stimulates the sympathetic nervous system which rises brown adipose tissue activity and the opposite was observed in a high salt intake (Coelho et al.) [31]. Moreover, it has been reported that, in high salt intake rats, energy expenditure was higher, while nonfasting leptin concentration was let down (Coelho et al.) [31]. The increase in the body weight in rats treated with plant extract suggested a possible interference of its compounds involved in various mechanisms of weight gain.

Salt intake is associated with the development of impaired kidney and liver function, independent of its effects on blood pressure. Thus, serum urea, creatinine, and transaminases identified as indicators of kidney or liver function have been evaluated in this study [32]. An increase in serum creatinine, serum urea concentrations, and transaminases (ALT and AST) activities were observed in the salt group (hypertensive group) as compared with the control group. The treatment with plant extract reduced the levels and the activities of these biomarkers in this study. The results may indicate the protective role of the extract in various tissues including the kidney and liver from high-salt-induced injuries [33]. Coadministration of salt and amlodipine, used as reference substances, has also prevented the increase in the serum biomarkers such as ALT and AST, creatinine, and urea.

Dyslipidemia observed in hypertensive rats resulted from high-salt feeding. It is well known that a high serum concentration of total cholesterol is a major predisposing risk factor for cardiovascular diseases [34]. Administration of the aqueous extract of *Ricinodendron heudelotii,* as well as amlodipine, markedly reduced serum total cholesterol, and LDL-cholesterol and enhanced the HDL-cholesterol significantly. This effect is attributed to its ability to slow down the lipid peroxidation process and enhance antioxidant enzyme activity [35].

According to previous studies, the possible mechanism by which high-salt diet-induced hypertension is the overgeneration of superoxide anions and other free radicals resulting in oxidative stress [36]. The antioxidants such as catalase (CAT), superoxide dismutase (SOD), glutathione, and malondialdehyde (MDA) constitute the major defense against ROS-induced oxidative damage [36].

In the present study, *R. heudelotii* reduced oxidative stress by increasing glutathione, catalase, superoxide dismutase, and nitrites level and preventing lipid peroxidation as compared to the hypertensive group indicating that the plant extract could prevent oxidative stress. Thus, this antioxidative effect or protective action against oxidative-induced damage on the cell could be due to the presence in the extract *R. heudelotii* of alkaloids, organic acids, and phenolic compounds, known for their antioxidant properties [37–42]. Since the mechanisms by which antioxidants act are diverse and sometimes specific, further antioxidant testing both in vitro and in vivo is required to clarify the modes of action.

Oxidative stress plays an important role in the progression of salt-sensitive hypertension and the accompanying end-stage renal damage [43]. Histological analysis of the kidney structure showed a tubular clarification and leucocytic infiltration and collagen deposition in the hypertensive group in the hypertensive group. Excessive salt intake alone has been reported, to be associated with renal fibrosis and glomerular and tubular necrosis in rats [44]. The administration of the aqueous extract of *R. heudelotii* (40 and 6 mg/kg) attenuated or/and restored the histological changes or renal lesions. This result indicates that the extract could have nephroprotective activity due to the antioxidant compounds, which could be partly responsible for the observed effect. It has been reported that antioxidants alleviate renal injury and improve kidney function by reducing oxidative damage and/or inflammation [45]. Thus, antioxidants have therapeutic properties to prevent kidney damage, increase renal hemodynamics, and lower blood pressure. Moreover, it has been reported that *R. heudelotti* is a valuable source of omega-3 fatty acids and antioxidant vitamins present at a higher level in the seeds [46, 47]. The presence of these compounds in this plant could also be responsible for the observed biological effects.

The results indicated that better effects of the extract are related to the lower dose (6 mg/Kg) used. Absorption of plant secondary metabolites into the bloodstream and uptake by target cells are essential for phytochemicals to exert biological activities [48]. As phytochemicals are recognized by the human body as xenobiotics, their presence in the human body is transient [49] and influenced by their physicochemical properties, while taking into account the interactions that could lead to significant effects. Thus, the better effects may be attributed to interactions between phytochemicals but further studies need to be done to elucidate these results.

## 5. Conclusion

This study shows that the aqueous extract stem bark of *Ricinodendron heudelotii* has the potential to improve salt-induced hypertension and reduce lipid peroxidation in Wistar rats. There is a need to research its possible preservation as the plant is seasonal. Further studies should be carried out to ascertain the mechanism of action through which it reduces blood pressure.

## Figures and Tables

**Table 1:** Characterization of the compounds found in the analyzed extracts of *R. heudelotii*.

No.	t_*R*_ (min)	Observed [M-H]^−^	Molecular formula	Error (ppm)	Fragment ions	Assigned identification
1	1.74	195.0513	C_6_H_12_O_7_	−1.27	129.0191, 99.0085, 75.0088, and 59.0140	Gluconic acid
2	2.04	191.0199	C_6_H_8_O_7_	−1.36	111.0085 and 87.0091	Citric acid
3	6.073	153.0192	C_7_H_6_O_4_	1.22	109.0289	Dihydroxybenzoic acid
4	8.81	137.0243	C_7_H_6_O_3_	0.74	108.0215, 92.0275, 81.0349, and 53.0407	3,4-Dihydroxybenzaldehyde
5	14.074	342.1702 (+)	C_20_H_23_NO_4_	−0.66	311.1278, 297.1119, 282.0884, 265.0854, 237.0909, and 222.0667	Magnoflorine
6	17.26	342.1702 (+)	C_20_H_23_NO_4_	−0.56	178.0858	Tetrahydroprotoberberine alkaloid
7	21.693	356.1859 (+)	C_21_H_25_NO_4_	−0.61	192.1013, 177.0777, 165.0901, and 148.0748	Tetrahydropalmatine
8	21.88	342.1708 (+)	C_20_H_23_NO_4_	−2.02	178.0861 and 151.0747	Tetrahydroprotoberberine alkaloid
9	24.677	187.0976	C_9_H_16_O_4_	−0.27	169.0860, 125.0967, 97.0657, and 57.0349	Nonanedioic acid
10	32.98	201.1134	C_10_H_18_O_4_	−0.76	139.1124, 111.0816, and 57.0355	Sebacic acid
11	40.54	329.2342	C_18_H_34_O_5_	−2.61	311.2200, 293.2098, 229.1440, 211.1336, and 171.1022	Trihydroxy-octadecenoic acid

**Table 2:** Effect of the aqueous extract of *R. heudelotii* on body weight in salt-induced hypertensive rats.

*Body weight (g)*						
	Doses (mg/kg)	Week 0	Week 1	Week 2	Week 3	Week 4
Control	–	151.0 ± 2.6	164.3 ± 5.8	168.4 ± 2.7	173.9 ± 2.9	174.3 ± 3.4
Salt	–	118.4 ± 4.2^c^	116.2 ± 1.9^c^	121.3 ± 2.5^c^	127.8 ± 1.8^c^	128.3 ± 0.7^c^
Salt + *R. heudelotii*	40	109.4 ± 4.0^c^	106.7 ± 6.0^c^	112.3 ± 4.2^c^	128.7 ± 2.4^c^	126.1 ± 5.0^c^
Salt + *R. heudelotii*	20	103.0 ± 2.9^c^	106.3 ± 3.6^c^	113.0 ± 3.2^c^	124.8 ± 4.3^c^	120.3 ± 4.4^c^
Salt + *R. heudelotii*	6	104.8 ± 2.5^c^	105.0 ± 3.4^c^	111.3 ± 3.8^c^	129.8 ± 3.1^c^	128.1 ± 2.7^c^
Salt + amlodipine	10	108.3 ± 3.6^c^	112.6 ± 4.6^c^	116.3 ± 4.6^c^	122.2 ± 4.7^c^	123.0 ± 4.5^c^

All values are expressed as mean ± SEM from 5 rats. ^c^*p* < 0.001 compared to the control. *R. heudelotii*, *Ricinodendron heudelotii*.

**Table 3:** Effect of the aqueous extract of *R. heudelotii* on relative organ weight in salt-induced hypertensive rats.

Relative organ weight (g/100 g of body weight)					
	Doses (mg/kg)	Liver	Kidneys	Heart	Aorta
Control	–	2.97 ± 0.06	0.60 ± 0.02	0.32 ± 0.01	0.10 ± 0.01
Salt	–	3.49 ± 0.04^c^	0.75 ± 0.02^c^	0.39 ± 0.01^b^	0.14 ± 0.11^a^
Salt + *R. heudelotii*	40	3.34 ± 0.02^a^	0.74 ± 0.03^b^	0.34 ± 0.01^*∗∗*^	0.10 ± 0.18
Salt + *R. heudelotii*	20	3.26 ± 0.09	0.71 ± 0.02^a^	0.33 ± 0.01^*∗∗∗*^	0.09 ± 0.18^*∗*^
Salt + *R. heudelotii*	6	3.03 ± 0.04^*∗∗∗*^	0.63 ± 0.01^*∗∗*^	0.33 ± 0.01^*∗∗∗*^	0.09 ± 0.18^*∗*^
Salt + amlodipine	10	3.40 ± 0.11^b^	0.72 ± 0.03^a^	0.38 ± 0.01^c^	0.12 ± 0.13

All values are expressed as mean ± SEM from 5 rats. ^a^*p* < 0.05, ^b^*p* < 0.01, and ^c^*p* < 0.001 compared to control and ^*∗*^*p* < 0.01, ^*∗∗*^*p* < 0.01, and ^*∗∗∗*^*p* < 0.001 compared to hypertensive group. *R. heudelotii*, *Ricinodendron heudelotii*.

**Table 4:** Effect of the aqueous extract of *Ricinodendron heudelotii* on biochemical parameters.

	Control	Salt + *R. heudelotii*				Salt + amlodipine
Parameters	–	–	40 mg/kg	20 mg/kg	6 mg/kg	10 mg/kg
CHL (mg/dl)	62.6 ± 2.5	97.4 ± 7.4^c^	72.2 ± 1.9^*∗∗*^	77.4 ± 6.7^*∗*^	76.4 ± 3.5^*∗∗*^	72.8 ± 4.8^*∗∗*^
HDL (mg/dl)	25.9 ± 2.2	16.0 ± 1.2^c^	17.9 ± 0.7	21.4 ± 0.6^*∗*^	21.9 ± 0.4^*∗*^	21.3 ± 1.2^*∗*^
LDL (mg/dl)	34.8 ± 1.5	61.7 ± 7.8^c^	41.9 ± 1.2^*∗∗*^	43.6 ± 5.1^*∗∗*^	41.6 ± 2.4^*∗∗*^	41.3 ± 3.4^*∗∗∗*^
Trig (mg/dl)	62.5 ± 2.9	95.2 ± 11.0	65.2 ± 3.9	70.4 ± 4.0	52.9 ± 4.8	64.3 ± 3.9
Prot (mg/dl)	81.4 ± 0.3	74.5 ± 0.3	75.0 ± 0.5	78.3 ± 0.2	81.1 ± 0.2	79.8 ± 0.2
Urea (mmol/l)	4.1 ± 0.4	15.0 ± 6.0^a^	5.7 ± 1.5^*∗*^	5.7 ± 1.8^*∗*^	5.8 ± 1.1^*∗*^	6.1 ± 1.0^*∗*^
Crea (mg/dl)	0.5 ± 0.1	1.7 ± 0.1^c^	0.9 ± 0.3^*∗∗*^	1.0 ± 0.0^*∗*^	1.0 ± 0.2^*∗*^	0.6 ± 0.0^*∗∗∗*^
ALT (U/l)	28.5 ± 4.3	82.21 ± 5.7^c^	47.0 ± 7.5^*∗*^	81.0 ± 19.1^b^	14.8 ± 1.9^*∗∗∗*^	58.6 ± 14.0
AST (U/l)	61.1 ± 14.6	204.7 ± 9.4^c^	58.4 ± 14.0^*∗∗∗*^	58.6 ± 12.0^*∗∗∗*^	49.2 ± 11.7^*∗∗∗*^	56.5 ± 10.8^*∗∗∗*^

All values are expressed as mean ± SEM from 5 rats. ^a^*p* < 0.05, ^b^*p* < 0.01, and ^c^*p* < 0.001 compared to control and ^*∗*^*p* < 0.01, ^*∗∗*^*p* < 0.01, and ^*∗∗∗*^*p* < 0.001 compared to the hypertensive group. CHL, total cholesterol; HDL, high-density lipoprotein; LDL, low-density lipoprotein; Pro, total protein; Trig, triglycerides; Crea, creatinine; ALT, alanine aminotransferase; AST, aspartate aminotransferase; *R. heudelotii*, *Ricinodendron heudelotii*.

## Data Availability

The data used to support the findings of this study can be obtained from the corresponding author upon request.
